# Dataset on the scale validation of Islamic piety

**DOI:** 10.1016/j.dib.2020.106360

**Published:** 2020-09-30

**Authors:** Raj Maham, Omar Khalid Bhatti

**Affiliations:** a Department of Business Administration, Iqra University, Islamabad Campus, Pakistan; b Department of Business, Istanbul Medipol University, Turkey

**Keywords:** Islamic piety, Islamic spirituality, Islamic social responsibility, Organizational Behavior

## Abstract

The data obtained from banking sector of Pakistan aims to enhance the understanding of the *Islamic Piety*. A semi-structure questionnaire was used to collect data from banking staff in Pakistan. The variable of the dataset is Islamic Piety which comprises of two sub- constructs that is; Islamic spirituality (IS) and Islamic social responsibility (ISR). Mohsen (2007) questionnaire for Islamic Piety was use and collected data from 500 employees. These employees were affiliated with the top nine credit rated banks of Pakistan in the federal and provincial capitals of the country. The data collected from the survey contributes itself to quantitative analysis using both descriptive as well as inferential statistics both of which are needed to respond to the research questions. The two sub-constructs are established based on the data.

## Specifications Table

**Table:** 

Subject	Psychological Science, Organizational Behavior.
Specific subject area	Islamic Piety in the Banking Sector of Pakistan.
Type of data	Table.
How data were acquired	The survey data adopted Mohsen (2007) questionnaires for this empirical research and used convenient sample of 500 employees working in the top nine credit rated banks of Pakistan located in the federal and provincial capitals of the country. All data was collected after consent of respondents.
Data format	Raw and Analysed data.
Parameters for data collection	Research population included all employees working in the nine selected banks of Pakistan.
Description of data collection	Convenient sampling was used to gain data through questionnaires. Respondent's questionnaires were vetted manually for missing or irrelevant values prior to data analysis. KMO, Bartlett, and Reliability tests were applied before factor analysis.
Data source location	Pakistani employees in the urban areas and provincial capitals.
Data accessibility	With the article.
Related research article	Maham, R., & Bhatti, O. K. (2019). Impact of Islamic piety (Islamic piety) on employee happiness: A study of Pakistan's banking sector. *Cogent Business & Management, 6*(1), 1678554. https://doi.org/10.1080/23311975.2019.1678554.

## Value of the Data

• The data was formerly collected to analyze the Impact of Islamic Piety on employee happiness in the banking sector of Pakistan.
• The Banking Sector was selected as it is deemed one of the most hectic and stressful working environment in context of Pakistan. Importantly the banking sector of Pakistan is also one of the most topped sectors among all businesses which thrived during the present times of economic crisis in Pakistan [1].
• The data from the present research can be employed for further research. As it can be used in future studies to understand Islamic Spirituality and Islamic Social Responsibility.
• The present Study and data also assist in extending the body of literature from Islamic Management perspective. The data from the present research can be employed to understand Islamic Spirituality and Islamic Social Responsibility, as well as assist in extending the body of literature from Islamic Management perspective.
• This research can also be replicated in other Muslim and Non-Muslim countries to better understand the behavior, motivation, and world view of Muslim employees.
• Researchers from diverse fields like; social psychology, psychology of religion, organizational behavior, ethics and religion, and human resource management can benefit from this data by conducting further experiments that may include other output variables (e.g., employee well-being, social responsibility, innovation and integrity). They can also examine the group differences based on cultural and demographic variables, including gender, age, and experience.
• The data also holds significance for Policy makers and organizational leaders as they can incorporate Islamic Piety construct in their general HR training modules which could help improving the overall effectiveness of their Muslim Employees.
• Islam is the second largest religion in the world with approximate population of 1.8 billion. The Muslim world spreads from Indonesia to Morocco. Therefore, the present data can also be used by other researchers and investigators to further improve the generalizability and validity of the scale of Islamic Piety.

## 1 Data Description

### 1.1 Demographic characteristics of respondents

Distribution frequency of the demographic variables of the respondents is shown in Table 1 below. The table shows a clear distinction in the gender of the respondents that included a majority of males at 70.8% compared to 29.2% of female respondents. The gender imbalance is expected in Pakistan, which is a male dominated society where women play a less active role in business. The age spectrum of the sample consisted 44% of respondents between the age of 25–29, 26% between the age of 30–34, 12.6% between the age of 35–39, 8.8% between the age of 20–24 and 8.6% between the age of 40 and above. The marital characteristics comprises of 51.2% married and 48.8% unmarried. The education qualification of the sample consists of 59.8% respondents having a master's degree, 24.2% have a bachelor's degree, and 14.6% have a degree in MS/MPhil while 1.4% have intermediate certificates. The sample's job category breakdown consists of 54.8%-line managers, 33.2% support staff, 11% middle managers and 1% top managers. The breakdown of number of years of experience in present position consists of 60% between 1 and 5 years, 30.4% less than 1 year, 7.8% between 6 and 10 years, and 1.8% more than 10 years. The breakdown for the total work experience in general consists of 14.2% less than 1 year, 45.8% between 1 and 5 years, 40% greater than 6 years and above. The breakdown of number of employees in company (bank branch) consists of 48.4% between 11 and 20 employees in company, 30.6% more than 20 employees in company, 31% between 1 and 10 employees in the company. All the respondent were Muslims. Table 4.3 below presents the distribution of respondents per sample characteristics.

**Table 1:** Distribution of Respondents Per Sample Characteristics.

Characteristics		N	%
Gender	Male	354	70.8
	Female	146	29.2
Age	20–24	44	8.8
	25–29	220	44.0
	30–34	130	26.0
	35–39	63	12.6
	40 and above	43	8.6
Marital status	Married	256	51.2
	Unmarried	244	48.8
Qualification	Intermediate	7	1.4
	Bachelors	121	24.2
	Master	299	59.8
	MS/MPHIL	73	14.6
Job designation	Support Staff	166	33.2
	Line Manager	274	54.8
	Middle Manager	55	11.0
	Top management	5	1.0
Experience in current position	Less than 1 year	152	30.4
	1–5 Years	300	60.0
	6–10 Years	39	7.8
	>10	9	1.8
Work experience in general	<1 year	71	14.2
	1–5 Years	229	45.8
	>6 years and above	200	40.0
Number of employees in company	1–10	105	21.0
	11–20	242	48.4
	>20	153	30.6

## 2 Experimental Design, Material and Methods

### 2.1 KMO, Bartlett, and reliability

During data collection, all necessary and important ethical considerations were ensured i.e. confidentiality, anonymity, and informed consent. In addition, the administration of questionnaires to respondents was based on their willingness to respond to the survey.

The present research to measure Islamic piety was adopted from the seminal work of Mohsen (2007). The same scale was first validated in Yamen and Malaysia. Since the scale was employed for the first time in Pakistan, hence, face and content validity were conducted on all the measurements. The items in the questionnaire were screened by two specialists from the field of organizational behavior and Islamic Management and three experts of Islamic studies. The comments and suggestions by the experts allowed to proceed with the scale in the same manner and order. Example items include “I supplicate Allah whenever I face difficulty in my work”, “Whenever I make a mistake I ask Allah's forgiveness”, “I treat my co-workers equally” and “I help my co-workers who need help”. Further, the design of the present scale also provides a stimulus for test taker response and the mechanism for response. As Islamic Spirituality and Islamic social responsibility measures the strength of individual differences in aspects related to Islamic Piety manifestations of Muslim employees, it is rated on a 7-point Likert-type scale (see appendix for complete questionnaire). According to the scholars, a wide range of scale may allow respondents to have enough flexibility in answering the questionnaire [3], [4], [5], [6], [7]. Hence the same was applied in the present study.

Notably, a survey design was used to collect data on Islamic Piety comprised of two components IS and ISR. Islamic Piety is defined as God's consciousness [2,3]. IS and ISR were measured by 18-items and 35-items scale, respectively. The internal consistency of the IS scale amounted to 0.894 and ISR scale amounted to 0.896. IS sub-components are Belief (*Iman*), Rituals (*Ibadat*), Repentance – seeking Allah's forgiveness and Remembrance of Allah. ISR sub-components are Patience, Emotional control, Forgiveness, Charity, Justice, Integrity, fulfillment of the covenant, Truthfulness, Love of family, and Guarding chastity [2], [3], [4].

All 12 sub-constructs were reviewed for their Cronbach's alpha values. Emotional control had a Cronbach's alpha value of 0.456 out of 1 and is not acceptable for inclusion in the analysis. Justice and guarding chastity are also questionable and hence excluded. Authors ran a factor analysis with principal component factoring and varimax rotation. In order to determine whether the factor analysis was appropriate for the data set, the analysis checked the Kaiser–Meyer–Olkin (KMO) measure of sampling adequacy and Bartlett's test of sphericity [6]. The KMO measure was in excess of 0.500 and made the data suitable for factor analysis. Moreover, Bartlett's test provided a highly significant chi-square statistic for Ability 0.000 and indicated adequate correlation between items. Thus, factor analysis was appropriate for the data set. One dimension of IS that is ritual is deleted and one dimension of ISR that is fulfillment of the covenant is also obsolete as the communality value do not fit well with the factor solution.

Discussing the EFA outputs, overall, 9 sub and 3 major factors were run for EFA indices. One EFA factor was generated for each sub factor which means that factor one is truly representing the overall variance of each variable. Over all 9 sub-constructs were finalized.

### 2.2 Confirmatory factor analysis

The AMOS software was used to conduct the confirmatory factor analysis (CFA) to validate constructs [7]. During the CFA, each construct is validated individually for first order. Table 2 presents the fitness test of all sub-constructs.

**Table 2:** Compatibility Tests.

Constructs	Absolute fit		Incremental fit	Parsimonious fit
	RMSEA	GFI	CFI	Chi sq/df
Ritual	0.112	0.971	0.935	36.128
Repentance	0.080	0.978	0.971	27.955
Remembrance	0.068	0.994	0.901	6.578
Patience	0.001	0.996	0.999	4.833
Forgiveness	0.426	1	1	000
Integrity	0.075	0.977	0.984	34.209
Truthfulness	0.451	1	1	000
Love of family	0.482	1	1	000
Charity	0.491	1	1	000

The analysis of the IS sub-construct provided a regression weighed estimate value of items below 0.60 and the value of all fitness index demonstrated that the fitness of this sub-construct model was achieved. All 14 items were retained in this sub-construct. The analysis of the ISR sub-construct also provided a regression weighed estimate value of items below 0.60 and the value of all fitness index demonstrated that the fitness of this sub-construct model was achieved. Therefore, all items were retained in this sub-construct also.

Table 2 shows compatibility test. The analysis show that the four sub constructs can be represented by their respective indicator variables that are repentance, remembrance, patience and integrity. The fitness test of CFA model found that all fitness index values [8], including Root Mean Square of Error Approximation (RMSEA), Goodness of Fit Index (GFI), Comparative Fit Index (CFI), and Chi Square / Degree of Freedom (Chi sq/df) of the four sub constructs are achieved.

Table 3 depicts the reliability and validity of the constructs after the confirmatory factor analysis.

**Table 3:** Reliability and Validity of Constructs.

Construct	Cronbach Alpha	Reliability constructs	Average variance extracted (AVE)
**Repentance**	0.696	0.818	49.4
**Remembrance**	0.764	0.852	59.2
**Patience**	0.680	0.807	51.2
**Integrity**	0.842	0.887	60.8

Table 4 explains the discriminant validity index the square root value of Average Variance Extracted (AVE) of Repentance, Remembrance, Patience and Integrity is greater than the correlation between Repentance, Remembrance, Patience and Integrity, then discriminant validity has been achieved.

**Table 4:** Discriminant Validity Index.

	Repentance	Remembrance	Patience	Integrity
**Repentance**	0.70			
**Remembrance**	.740^⁎⁎^	0.77		
**Patience**	.551^⁎⁎^	.518^⁎⁎^	0.72	
**Integrity**	.155^⁎⁎^	.248^⁎⁎^	0.078	0.78

## Ethics Statement

All procedures performed in this research followed the ethical standards of the institutional and/or national research committee and with the 1964 Helsinki declaration and its later amendments or comparable ethical standards. The participation by the respondents in the data collection was voluntary without any compulsion. Informed consent of all participants was obtained. Confidentiality and anonymity of respondents and responses was ensured.

## Declaration of Competing Interest

The authors declare that they have no known competing financial interests or personal relationships which have, or could be perceived to have, influenced the work reported in this article.

## Appendix Supplementary materials

Image, application 1

Image, application 2
