# Supplementary material for: Dataset on the scale validation of Islamic piety
Source: Data Brief. 2020 Sep 30;33:106360. doi: 10.1016/j.dib.2020.106360 (PMC7560710; doi:10.1016/j.dib.2020.106360)
Supplement: Supplementary file 1 [file mmc1.docx]

**Questionnaire**

***SECTION A: Islamic Spirituality***

Please read carefully and circle the number that best represents your behavior from the following statements, according to this criterion:

| 1. | Statement | Strongly  Disagree | Disagree | Somewhat  Disagree | Neutral | Somewhat  Agree | Agree | Strongly  Agree |
| --- | --- | --- | --- | --- | --- | --- | --- | --- |
|  |  | 1 | 2 | 3 | 4 | 5 | 6 | 7 |

| 1. | Whenever possible, I encourage my co-workers to visit the prayer rooms for prayers (rituals) | 1 | 2 | 3 | 4 | 5 | 6 | 7 |
| --- | --- | --- | --- | --- | --- | --- | --- | --- |
| 2. | I inspire my co-workers to fast and breakfast collectively (rituals) | 1 | 2 | 3 | 4 | 5 | 6 | 7 |
| 3. | I practice optional fasting (remembrance of Allah) | 1 | 2 | 3 | 4 | 5 | 6 | 7 |
| 4. | I encourage my co-workers to pray together at work (rituals) | 1 | 2 | 3 | 4 | 5 | 6 | 7 |
| 5. | When I am confronted with competing alternatives in decision making, I perform *istikhara* prayer (rituals) | 1 | 2 | 3 | 4 | 5 | 6 | 7 |
| 6. | Whenever I pay my *zakat*, I make sure I calculate it correctly (rituals) | 1 | 2 | 3 | 4 | 5 | 6 | 7 |
| 7. | I ask Allah to help me when I make important decisions at my work (decision) (repentance) | 1 | 2 | 3 | 4 | 5 | 6 | 7 |
| 8. | I supplicate Allah whenever I face difficulty in my work (belief) | 1 | 2 | 3 | 4 | 5 | 6 | 7 |
| 9. | Whenever I make a mistake, I ask Allah’s forgiveness (remembrance) | 1 | 2 | 3 | 4 | 5 | 6 | 7 |
| 10. | I do my best to perform all five prayers regardless of how busy I am  (remembrance of Allah) | 1 | 2 | 3 | 4 | 5 | 6 | 7 |
| 11. | I do my duties in the best way I could and leave the outcomes to be determined by Allah (remembrance of Allah) | 1 | 2 | 3 | 4 | 5 | 6 | 7 |
| 12. | I do my best in my work because Allah is watching me  (remembrance of Allah) | 1 | 2 | 3 | 4 | 5 | 6 | 7 |
| 13. | I ask forgiveness from my co-workers that I have wronged (repentance) | 1 | 2 | 3 | 4 | 5 | 6 | 7 |
| 14. | I deal with co-workers with justice and generosity (repentance) | 1 | 2 | 3 | 4 | 5 | 6 | 7 |
| 15. | I direct my dedication to Allah alone (belief) | 1 | 2 | 3 | 4 | 5 | 6 | 7 |
| 16. | I stay away from haram acts in my work to avoid Allah’s divine wrath (repentance) | 1 | 2 | 3 | 4 | 5 | 6 | 7 |
| 17. | I apologize for my mistakes when I realize them at work (repentance) | 1 | 2 | 3 | 4 | 5 | 6 | 7 |
| 18. | If any coworker would like to for *Hajj* I will try to help him getting paid leave (rituals) | 1 | 2 | 3 | 4 | 5 | 6 | 7 |

***Section B: Islamic Social Responsibility***

Please read carefully and circle the number that best represents your behavior from the following statements, according to this criterion:

| 1. | Statement | Strongly  Disagree | Disagree | Somewhat  Disagree | Neutral | Somewhat  Agree | Agree | Strongly Agree |
| --- | --- | --- | --- | --- | --- | --- | --- | --- |
|  |  | 1 | 2 | 3 | 4 | 5 | 6 | 7 |

| 1. | When I promise my co-workers, I fulfill my promise (covenant) | 1 | 2 | 3 | 4 | 5 | 6 | 7 |
| --- | --- | --- | --- | --- | --- | --- | --- | --- |
| 2. | I mind to see a co-worker not being honest (integrity) | 1 | 2 | 3 | 4 | 5 | 6 | 7 |
| 3. | I encourage my co-workers to fulfill their promises (covenant) | 1 | 2 | 3 | 4 | 5 | 6 | 7 |
| 4. | I abide by agreements I make with my co-workers (covenant) | 1 | 2 | 3 | 4 | 5 | 6 | 7 |
| 5. | Before making a decision, I wait until my co-worker’s finish expressing their opinions (patience) | 1 | 2 | 3 | 4 | 5 | 6 | 7 |
| 6. | I am not afraid to tell the truth (truthfulness) | 1 | 2 | 3 | 4 | 5 | 6 | 7 |
| 7. | I tell the truth regardless of the consequences (truthfulness) | 1 | 2 | 3 | 4 | 5 | 6 | 7 |
| 8. | I tend to be more forgiving with my co-workers (forgiveness) | 1 | 2 | 3 | 4 | 5 | 6 | 7 |
| 9. | I accept excuses from my co-workers (forgiveness) | 1 | 2 | 3 | 4 | 5 | 6 | 7 |
| 10. | I encourage my co-workers to be honest (truthfulness) | 1 | 2 | 3 | 4 | 5 | 6 | 7 |
| 11. | I find it wrong when my colleagues have relations beyond wedlock (chastity) | 1 | 2 | 3 | 4 | 5 | 6 | 7 |
| 12. | I speak negative of my co-workers behind their back (backbiting) (integrity) | 1 | 2 | 3 | 4 | 5 | 6 | 7 |
| 13. | I act against co-workers out of revenge (emotional control) | 1 | 2 | 3 | 4 | 5 | 6 | 7 |
| 14. | I meddle in my co-worker’s personal affairs (integrity) | 1 | 2 | 3 | 4 | 5 | 6 | 7 |
| 15. | I use organizational resources for my personal use (integrity) | 1 | 2 | 3 | 4 | 5 | 6 | 7 |
| 16. | I manipulate my co-workers (integrity) | 1 | 2 | 3 | 4 | 5 | 6 | 7 |
| 17. | I take the credit for my co-workers’ ideas (integrity) | 1 | 2 | 3 | 4 | 5 | 6 | 7 |
| 18. | I burden my co-workers with workloads (justice) | 1 | 2 | 3 | 4 | 5 | 6 | 7 |
| 19. | I easily get angry for minor reasons (emotional control) | 1 | 2 | 3 | 4 | 5 | 6 | 7 |
| 20. | Co-workers willingly approach me for my judgment during dispute (patience) | 1 | 2 | 3 | 4 | 5 | 6 | 7 |
| 21. | I treat my co-workers equally (justice) | 1 | 2 | 3 | 4 | 5 | 6 | 7 |
| 22. | I look for opportunities to be of service to my co-workers (justice) | 1 | 2 | 3 | 4 | 5 | 6 | 7 |
| 23. | I try my best to be generous (*ihsan*) to co-workers as possible (sadaqah) | 1 | 2 | 3 | 4 | 5 | 6 | 7 |
| 24. | I have the passion to offer help to co-workers purely for the sake of Allah  (sadaqah) | 1 | 2 | 3 | 4 | 5 | 6 | 7 |
| 25. | In case of conflict between me and co-workers, I try to settle it on my own (emotional control) | 1 | 2 | 3 | 4 | 5 | 6 | 7 |
| 26. | I am patient in negotiations (patience) | 1 | 2 | 3 | 4 | 5 | 6 | 7 |
| 27. | I forgive my co-workers even if they hurt me on purpose (forgiveness) | 1 | 2 | 3 | 4 | 5 | 6 | 7 |
| 28. | When a co-worker hurts me, I reciprocate with kindness (patience) | 1 | 2 | 3 | 4 | 5 | 6 | 7 |
| 29. | I help my co-workers who need help (sadaqah) | 1 | 2 | 3 | 4 | 5 | 6 | 7 |
| 30. | I take responsibility of the family's happiness while remaining steadfast to achievement (love of family) | 1 | 2 | 3 | 4 | 5 | 6 | 7 |
| 31. | I aim to properly prioritize between career and lifestyle (love of family) | 1 | 2 | 3 | 4 | 5 | 6 | 7 |
| 32. | I take action effectively to manage work-life balance (love of family) | 1 | 2 | 3 | 4 | 5 | 6 | 7 |
| 33. | I strive to effectively manage relationships with the opposite sex in the organization (chastity) | 1 | 2 | 3 | 4 | 5 | 6 | 7 |
| 34. | I avoid any illegal relationships and all kinds of sins (chastity) | 1 | 2 | 3 | 4 | 5 | 6 | 7 |
| 35. | I get irritated easily when someone criticize me (emotional control) | 1 | 2 | 3 | 4 | 5 | 6 | 7 |
